# How does DRG reform affect length of stay and hospitalization costs in traditional Chinese medicine hospitals?—an empirical analysis using interrupted time-series model from two cities in western China

**DOI:** 10.3389/fpubh.2026.1762327

**Published:** 2026-02-04

**Authors:** Wei Zhang, Li-Jun Zhong, Sheng-Cong Tao, Meng-en Chen

**Affiliations:** 1 School of Health Management, Gansu University of Chinese Medicine, Lanzhou, China; 2 Lanzhou Health Statistics Information Center, Lanzhou Health and Wellness Commission, Lanzhou, China; 3 School of Traditional Chinese Medicine, Beijing University of Chinese Medicine, Beijing, China; 4 School of Management, Beijing University of Chinese Medicine, Beijing, China

**Keywords:** DRG, hospitalization costs, ITS, length of stay, TCM hospitals

## Abstract

**Objectives:**

China is advancing Diagnosis Related Group (DRG)-based health insurance reforms to address rising healthcare costs, with traditional Chinese medicine (TCM) hospitals as a key focus. Evaluating the impact of TCM DRG reforms is crucial for China to leverage its unique medical practices in reducing the economic burden of disease.

**Methods:**

We collected 535,886 hospitalization records regarding length of stay and hospitalization costs from TCM hospitals in Qingyang City and Tianshui City, Gansu Province, for the period from January 2017 to June 2022 (313,823 from Qingyang and 222,062 from Tianshui). A comparative analysis of the DRG reform’s implementation effects in secondary and tertiary TCM hospitals in Qingyang was conducted using descriptive statistics and two groups interrupted time-series (ITS) model.

**Results:**

In Qingyang’s secondary TCM hospitals, there were no significant changes in the length of stay and hospitalization costs before the DRG reform (*p* > 0.05). However, post-reform, both metrics exhibited a notable increase, averaging 0.02 days and CNY 34.54 per month (*p* < 0.05). Conversely, the length of stay in tertiary TCM hospitals showed no significant changes before the reform (*p* > 0.05), while hospitalization costs exhibited a significant upward trend (*p* < 0.05). After the implementation of the DRG reform, both length of stay and hospitalization costs significantly declined, with monthly reductions of 0.06 days and CNY 60.47 (*p* < 0.05).

**Conclusion:**

DRG reform has positively influenced the length of stay and hospitalization costs in TCM hospitals, with tertiary facilities showing better outcomes than secondary ones. To improve the effectiveness of DRG health insurance payment reforms in China, it is essential to enhance the qualifications of medical personnel and advance information technology infrastructure in TCM hospitals. Furthermore, implementing differentiated reforms across TCM hospitals and strengthening the systematic development of health insurance payment structures are critical.

## 1 Introduction

The global disease burden continues to escalate, with financial pressures in some regions undermining population health protection capabilities (1). People also show a sense of helplessness in maintaining their overall health, particularly when facing severe and high-burden diseases such as tumors. In China, total healthcare expenditure rose from 2.8119 trillion RMB in 2012 to 8.5327 trillion RMB in 2022. Per capita healthcare spending increased from 2,068.76 RMB to 6,044.09 RMB, a nearly threefold rise. The basic medical insurance premiums paid by individuals have grown year by year, while the pressure from out-of-pocket medical expenses has been steadily increasing (2). As the aging population in China continues to grow, the future economic burden of diseases is expected to become even more substantial (3, 4). Ultimately, finding effective strategies to control healthcare cost growth while maintaining the quality of medical services has become a critical public health issue for low- and middle-income countries, including China, as well as for the global community.

The concept of DRG was first proposed by Professors Fetter and Thompson at Yale University (5). DRG is a case grouping model developed based on the consumption of medical resources, designed for classifying clinical medical services and facilitating healthcare cost payments. Cases grouped under the same DRGs demonstrate clinical consistency and relatively uniform resource consumption (6). Since the implementation of the DRG payment system in the United States in 1983, the growth rate of total hospital costs covered by Medicare has decreased significantly, from 18.5% in 1983 to 5.7% in 1990. Similarly, the growth rate of surgical fees declined from 14.5% in 1984 to 6.6% in 1992, with the average length of stays falling from 10.4 days in 1980 to 8.7 days in 1990, ultimately reducing to 6.7 days by 1995 (7). Given the substantial cost control advantages of the DRG system and its close integration with hospital management performance evaluations, the DRG payment system has been rapidly adopted by various countries, yielding notable results in healthcare reform (8–11). In the 1980s, the DRG management concept was introduced to China. Following 2000, China began developing the BJ-DRG system, which was pilot-tested in the Beijing area starting in 2011 (12). Subsequently, other DRG systems, including C-DRG and CN-DRG, were developed (13). In 2019, China’s National Healthcare Security Administration issued the “China Healthcare Security Diagnosis Related Groups (CHS-DRG) Scheme,” introducing DRG payments in 30 pilot regions with the goal of nationwide implementation in hospitals by 2025 (14, 15). This initiative marked the establishment of a national-level DRG payment system in China and signaled the beginning of significant reforms in DRG payment methodologies.

TCM is distinguished by its simplicity, convenience, effectiveness, and affordability, providing distinct advantages in the prevention and treatment of various diseases (16–18). As an integral component of China’s healthcare system, TCM also represents a significant focus of the country’s DRG healthcare payment reform. TCM into value-based payment systems (e.g., DRG/DIP) poses a global challenge due to the inherent tension between its holistic practice and standardized payment models. Although the WHO promotes its inclusion for universal health coverage, designing cost-effective mechanisms that recognize TCM’s unique value remains a key research gap. China’s ongoing DRG/DIP reforms in TCM provide a critical real-world context for advancing this international agenda.

This study aimed to investigate the impact of DRG payment reform on length of stay and hospitalization costs in TCM hospitals. By conducting a comparative analysis of data from TCM hospitals of various levels before and after the implementation of the DRG reform, this research sought to elucidate the effects and challenges associated with applying the DRG payment model in TCM settings. The findings clearly demonstrate the tangible effects of DRG reform on care efficiency and hospitalization costs within traditional Chinese medicine hospitals, offering valuable insights for the Chinese government in leveraging the unique strengths of Chinese medicine to address the persistent rise in healthcare costs.

## 2 Methods

### 2.1 Study design

To effectively evaluate the impact of DRG reform on TCM hospitals, this study employed a quasi-experimental design. The TCM hospitals in Qingyang City, Gansu Province, which implemented DRG reform, served as the pilot group, while the TCM hospitals in Tianshui City, Gansu Province, which has similar economic, social, and healthcare development levels but has not adopted DRG reform, were designated as the control group. A quasi-experimental model utilizing ITS analysis was employed to compare length of stay and hospitalization costs for inpatients at both hospitals, facilitating a comprehensive assessment of the impact of DRG reform on TCM hospitals.

Regarding the assumptions of ITS in the context of a non-randomized policy rollout and their application in this study, we have specifically addressed and validated the following key assumptions. Absence of concurrent confounding policies: Through a review of policy documentation, we confirmed that no other major healthcare payment reforms were introduced during the study period. The impact of the COVID-19 pandemic was controlled for by including a dummy variable in the model, and all statistical analyses and figures have been updated accordingly. Data continuity: Monthly hospitalization data were collected consistently and reliably throughout the observation period. Appropriateness of model specification: By ensuring data quality and performing correlation-based adjustments, the robustness of the results has been verified.

Additionally, due to the varying qualifications among TCM hospitals in China—evaluated based on factors such as hospital size, research focus, technical expertise, and medical equipment—significant disparities exist in the overall capabilities of hospitals at different levels. To mitigate potential bias arising from these differences in hospital classification, this study examined the effects of DRG reform on length of stay and hospitalization costs specifically for secondary and tertiary TCM hospitals, respectively.

### 2.2 Data sources

The data for our study were obtained from the Gansu Provincial Health and Wellness Commission. The commission provided hospitalization case data for all TCM hospitals in Qingyang (seven secondary TCM hospitals and one tertiary TCM hospital) and Tianshui (six secondary TCM hospitals and one tertiary TCM hospital) from January 2017 to June 2022, encompassing a total of 535,886 valid cases (313,823 from Qingyang and 222,062 from Tianshui). Professional technical personnel from the commission systematically extracted information regarding the length of stay and hospitalization costs for inpatients at TCM hospitals in both cities. This included basic statistical metrics such as average hospitalization costs, lengths of stay, and quartile values. It is important to highlight that the Gansu Provincial Health and Wellness Commission conducts regular data validation and cleaning for TCM hospitals within the system, as well as compiles and refines the data, thereby ensuring the quality of the data and the validity of the research.

The DRG healthcare payment system commenced its pilot operation in Qingyang, Gansu Province, in October 2019. Consequently, the period from January 2017 to September 2019 was designated as the pre-DRG reform phase, while the period from October 2019 to June 2022 was categorized as the post-DRG reform phase. Given that the research data pertained to healthcare economic costs, we utilized 2016 as the base year to adjust the relevant costs according to the Consumer Price Index (CPI) for healthcare services in Gansu Province from 2017 to 2022, thereby minimizing potential research bias.

### 2.3 Statistical analysis

Our study utilized two groups ITS model, a quasi-experimental design widely employed in public health and policy reform (19–23). The equation for the model is expressed as follows:


$$\begin{array}{l} Y_{t}=\beta_{0}+\beta_{1}T_{t}+\beta_{2}X_{t}+\beta_{3}X_{t}T_{t}+\beta_{4}Z+\beta_{5}ZT_{t}+\\ \beta_{6}ZX_{t}+\beta_{7}ZX_{t}T_{t}+\beta_{8}X_{\text{Covid}-19}+\varepsilon_{t} \end{array}$$

**Figure 1:**
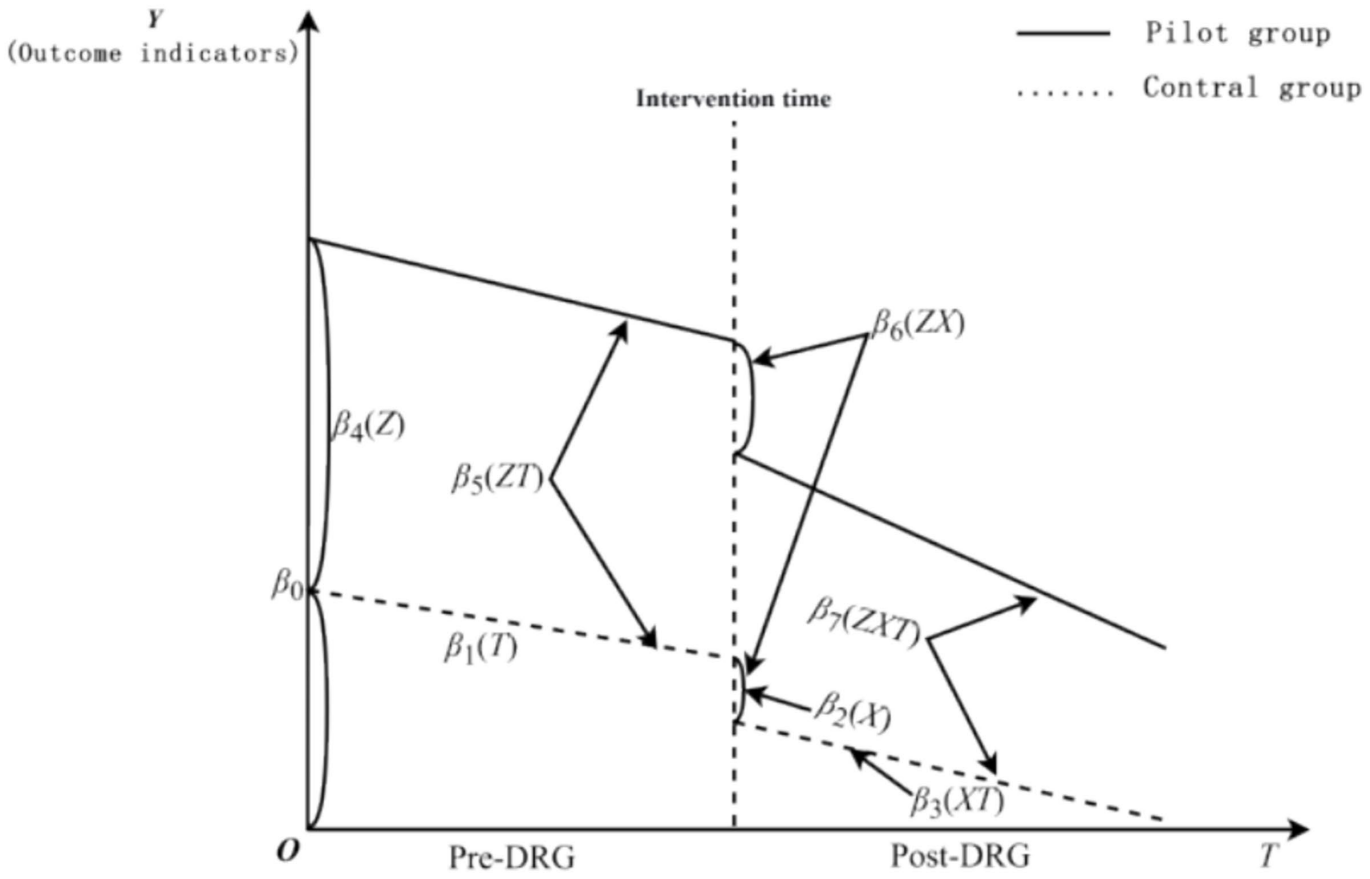
Schematic diagram of ITS model.

In this equation, $Y_{t}$ represents the dependent variable, which serves as the primary outcome indicator of the study. $\beta_{0}\;$ denotes the intercept for the control group, or the constant term. $\beta_{1}$ indicates the trend of changes in the control group before the reform, corresponding to its slope. $\beta_{2}\;$ reflects the level change in the control group at the time of the reform. $\beta_{3}$ represents the difference in slopes of the control group before and after the reform. $\beta_{4}$ indicates the difference in levels between the two groups before the reform, while $\beta_{5}$ signifies the difference in slopes between the two groups before the reform. $\beta_{6}$ captures the difference in level changes between the two groups at the time of the reform, measuring the short-term effects of the policy reform. Furthermore, $\beta_{7}$ refers to the difference in changes of slopes between the two groups before and after the reform, which allows for the assessment of long-term trends post-reform. Besides, $X_{t}$ , $X_{\text{Covid}-19}$ , and *Z* are dummy variables representing the DRG reform (with a value of “0” before the reform and “1” after), COVID-19 (with a value of “0” before the COVID-19 and “1” after) and cities (with Qingyang City coded as “1” and Tianshui City coded as “2”). The variable $T_{t}$ represents the time series, which is a continuous variable over the observation period of this study, spanning from January 2017 to June 2022. Since this study includes data from 66 consecutive months, the range of T is from 1 to 66. Moreover, the model also includes interaction terms $X_{t}T_{t}$ , $ZT_{t}$ , $ZX_{t}$ , and $ZX_{t}T_{t}$ , with $\varepsilon_{t}$ denotes the random error term. Given that the data on length of stay and hospitalization costs are skewed, we selected the monthly median values as the original data for the ITS model analysis to ensure the objectivity and rigor of the data analysis. A schematic diagram of the model is presented in Figure 1.

In addition, we utilized the Cumby-Huizinga test to evaluate the autocorrelation of the dependent variable (24). Adjustments and calculations were conducted using the “lag (#)” command in conjunction with the Newey-West method to mitigate potential research bias (25, 26). All statistical analyses were performed using Stata 15.0 software, with a significance level established at *α* = 0.05.

## 3 Results

### 3.1 Overview of length of stay and hospitalization costs in TCM hospitals across two cities

In Qingyang City’s secondary TCM hospitals, the length of stay for inpatients remained relatively stable from 2017 to 2019, followed by a slight increase from 2020 to 2022. Hospitalization costs, on the other hand, demonstrated a consistent upward trend throughout the entire period from 2017 to 2022. In contrast, the length of stay for inpatients in Tianshui City’s secondary TCM hospitals exhibited overall stability, with a minor upward trend observed from 2017 to 2022, while hospitalization costs remained stable, showing a slight decline during the same period. Detailed results are presented in Table 1.

**Table 1:** Length of stay and hospitalization costs of patients in secondary/tertiary TCM hospitals in Qingyang City and Tianshui City, 2017–2022.

Cities	Year	Secondary TCM hospitals		Tertiary TCM hospitals	
		Length of stay(days)/M (P_25_, P_75_)*	Hospitalization costs (CNY)/M (P_25_, P_75_)*	Length of stay(days)/M (P_25_, P_75_)*	Hospitalization costs (CNY)/M (P_25_, P_75_)*
Qingyang City	2017	7 (5, 9)	1835.87 (1274.00, 2640.00)	11 (7, 17)	5492.00 (3594.97, 7958.22)
	2018	7 (5, 8)	2005.00 (1395.00, 2848.79)	12 (7, 17)	6499.84 (4350.50, 9190.73)
	2019	7 (5, 9)	2228.66 (1607.37, 2978.12)	11 (7, 16)	6977.54 (4568.19, 9931.44)
	2020	7 (6, 9)	2219.66 (1663.90, 3139.29)	11 (7, 16)	6959.04 (4751.31, 10115.00)
	2021	7 (6, 9)	2994.69 (2166.52, 3719.26)	10 (7, 15)	6486.21 (4430.61, 9316.72)
	2022	7 (6, 9)	2987.69 (2077.78, 3736.11)	10 (7, 14)	6163.52 (4221.82, 8684.28)
Tianshui City	2017	7 (5, 10)	2690.15 (1915.00, 3737.40)	11 (8, 14)	6372.50 (4232.75, 8384.25)
	2018	8 (5, 11)	1957.41 (944.47, 3420.29)	11 (7, 14)	6608.00 (4161.31, 8832.84)
	2019	8 (6, 10)	2927.20 (1631.38, 3908.79)	10 (7, 13)	6472.74 (4069.71, 8650.18)
	2020	8 (5, 11)	3062.15 (1809.42, 4278.19)	10 (7, 13)	6484.72 (4405.15, 8419.82)
	2021	8 (5, 11)	2945.08 (1779.00, 3898.71)	10 (7, 13)	5989.29 (3863.19, 7856.58)
	2022	8 (6, 11)	3037.43 (2000.00, 4070.85)	9 (7, 12)	5945.55 (4171.56, 7605.68)

*M (P_25_, P_75_): median (the first quartile, the third quartile).

In tertiary TCM hospitals in Qingyang City, the length of stay for inpatients remained relatively stable from 2017 to 2019, followed by a noticeable downward trend from 2020 to 2022. Correspondingly, hospitalization costs showed a significant increase between 2017 and 2019, but declined markedly from 2020 to 2022. Similarly, in Tianshui City’s tertiary TCM hospitals, the length of stay exhibited an overall downward trend from 2017 to 2022, accompanied by a gradual decline in hospitalization costs over the same period (Table 1).

### 3.2 ITS analysis of DRG reform on length of stay and hospitalization costs in secondary TCM hospitals

We performed Cumby-Huizinga autocorrelation tests on length of stay and hospitalization costs in secondary TCM hospitals. The results suggested the presence of first-order autocorrelation in length of stay, while hospitalization costs displayed indications of second-order autocorrelation, as shown in Table 2. To ensure the validity of the ITS analysis, we accounted for autocorrelation effects by applying the ‘lag (1)/lag (2)’ command in our analysis.

**Table 2:** Autocorrelation test results of length of stay and hospitalization costs in secondary TCM hospitals.

Length of stay								Hospitalization costs							
H_0_: *q* = 0 (serially uncorrelated)				H_0_: *q* = lag-1				H_0_: *q* = 0 (serially uncorrelated)				H_0_: *q* = lag-1			
H_1_: s.c. present at range specified				H_1_: s.c. present at lag specified				H_1_: s.c. present at range specified				H_1_: s.c. present at lag specified			
lags	chi^2^	df	*p*-value	lags	chi^2^	df	*p*-value	lags	chi^2^	df	*p*-value	lags	chi^2^	df	*p*-value
1–1	7.51	1	0.006	1	7.51	1	0.006	1–1	34.60	1	<0.001	1	34.60	1	<0.001
1–2	14.64	2	0.001	2	3.49	1	0.062	1–2	35.39	2	<0.001	2	9.26	1	0.002
1–3	15.57	3	0.001	3	3.96	1	0.047	1–3	37.39	3	<0.001	3	0.60	1	0.437
1–4	18.89	4	0.001	4	3.68	1	0.055	1–4	38.33	4	<0.001	4	0.60	1	0.437
1–5	19.23	5	0.002	5	0.51	1	0.475	1–5	38.42	5	<0.001	5	0.29	1	0.588
1–6	21.72	6	0.001	6	0.46	1	0.497	1–6	38.74	6	<0.001	6	0.03	1	0.859

**Figure 2:**
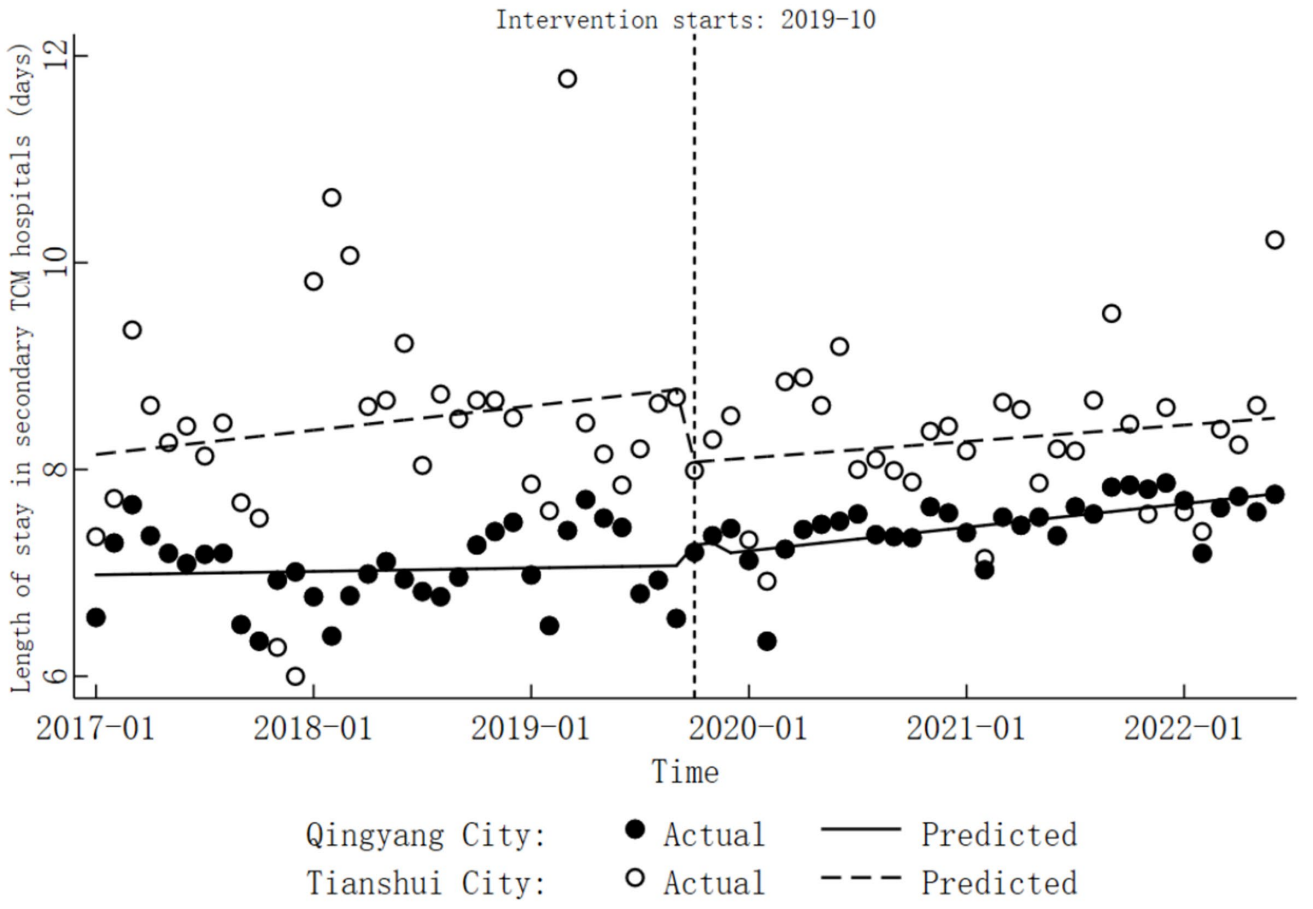
Trends in length of stay before and after the DRG reform in secondary TCM hospitals.

In Tianshui City’s secondary TCM hospitals, the trend in length of stay before DRG reform was not significant ( $\beta_{1}=0.02,$ *p* > 0.05). During the reform period, there was no significant change in length of stay ( $\beta_{2}=-0.72,$ *p* > 0.05). Furthermore, the trend after the reform also remained insignificant ( β=0.01, *p* > 0.05). Detailed results are provided in Tables 3, 4, with specific trend changes illustrated in Figure 2.

**Table 3:** Results of ITS analysis of length of stay and hospitalization costs in secondary TCM hospitals.

Variables	Length of stay					Hospitalization costs				
	Coefficient	Newey-west std. err	*t*-value	*p*-value	95% conf. interval	Coefficient	Newey-west std. err	*t*-value	*p*-value	95% conf. interval
*T*	0.02	0.02	1.14	0.256	[−0.01, 0.05]	4.24	12.07	0.35	0.726	[−19.65, 28.12]
*Z*	−1.28	0.43	−2.95	0.004	[−2.13, −0.42]	−565.31	325.18	−1.74	0.085	[−1208.98, 78.37]
*ZT*	−0.02	0.02	−0.87	0.386	[−0.06, 0.02]	11.92	12.92	0.92	0.358	[−13.65, 37.48]
*X*_2019m10_	−0.72	0.41	−1.74	0.084	[−1.53, 0.10]	433.66	226.80	1.91	0.058	[−15.28, 882.60]
*XT*_2019m10_	−0.01	0.02	−0.27	0.784	[−0.05, 0.04]	−9.57	12.83	−0.75	0.457	[−34.98 15.83]
*ZX*_2019m10_	0.92	0.45	2.06	0.042	[0.04, 1.80]	−722.41	248.26	−2.91	0.004	[−1213.83, −230.99]
*ZXT*_2019m10_	0.02	0.03	0.88	0.380	[−0.03, 0.07]	27.96	15.31	1.83	0.07	[−2.36, 58.27]
COVID-19	−0.11	0.14	−0.84	0.403	[−0.38, 0.15]	−13.77	118.79	−0.12	0.908	[−348.90, 221.37]
cons	8.26	0.40	20.67	<0.001	[7.47, 9.05]	2736.99	315.53	8.67	<0.001	[2112.41, 3361.57]

**Table 4:** Trends in hospitalization costs and length of stay in secondary TCM hospitals in Qingyang City and Tianshui City after DRG reform.

Indicators	Cities	Coefficient	Std. err.	*t*-value	*p*-value	95% conf. interval
Length of stay	Qingyang City	0.02	0.01	2.91	0.004	[0.01, 0.03]
	Tianshui City	0.01	0.02	0.86	0.391	[−0.02, 0.04]
	Difference	0.01	0.02	0.34	0.733	[−0.03, 0.04]
Hospitalization costs	Qingyang City	34.54	6.96	4.96	<0.001	[20.76, 48.32]
	Tianshui City	−5.34	4.59	−1.16	0.247	[−14.42, 3.75]
	Difference	39.87	8.34	4.78	<0.001	[23.37, 56.38]

The trend in the length of stay at Qinyang City’s secondary TCM hospitals before the implementation of DRG reform was not statistically significant (combined $\beta_{1}$ , $\beta_{5}$ ). Additionally, the change in the level of length of stay of the reform was also not significant (combined $\beta_{2}$ , $\beta_{6}$ ). However, following the reform, a significant upward trend was observed ( β=0.02, *p* < 0.05). Apart from this, the COVID-19 pandemic did not have a significant impact on the length of stay( $\beta_{8}=-0.11,$ *p* > 0.05). For detailed results, please refer to Tables 3, 4, and for specific trend changes, see Figure 2.

**Figure 3:**
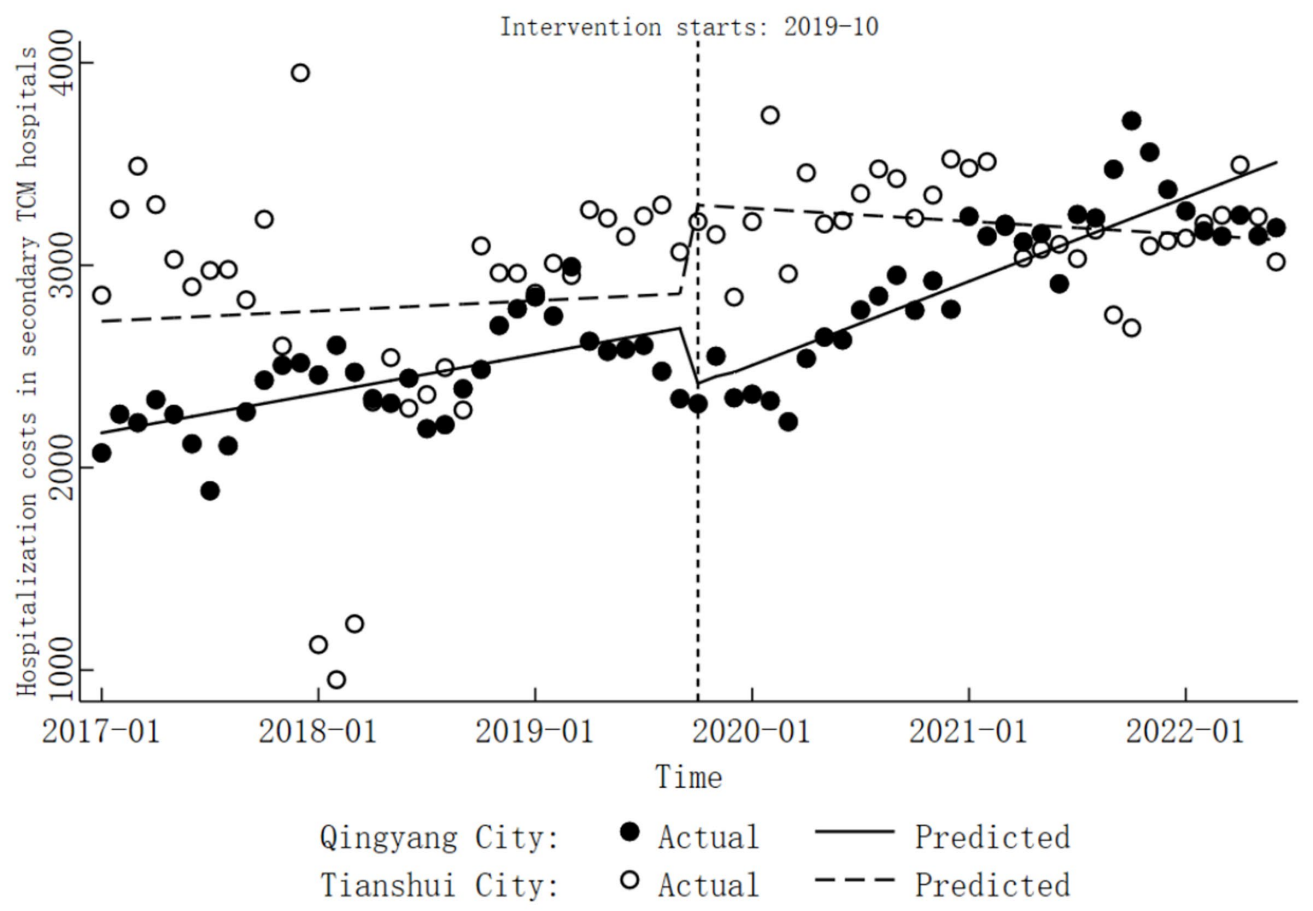
Trends in hospitalization costs before and after the DRG reform in secondary TCM hospitals.

In Tianshui City’s secondary TCM hospitals, the trend of hospitalization costs before DRG reform was not statistically significant ( $\beta_{1}=4.24,$ *p* > 0.05). During the reform period, no significant change was observed in hospitalization costs ( $\beta_{2}=433.66,$ *p* > 0.05). Following the reform, the trend of changes remained statistically insignificant ( β=−5.34, *p* > 0.05). Detailed results are presented in Tables 3, 4, while specific trend changes are illustrated in Figure 3.

The trend in hospitalization costs at secondary TCM hospitals in Qingyang City before the implementation of DRG reform was not statistically significant (comprehensive $\beta_{1}$ , $\beta_{5}$ ). During the reform period, there was also no significant change in hospitalization costs (combined $\beta_{2}$ , $\beta_{6}$ ). However, following the reform, a notable upward trend emerged, with an average monthly increase of CNY 34.54 ( β=34.54, *p* < 0.05). Apart from this, the COVID-19 pandemic did not have a significant impact on hospitalization costs ( $\beta_{8}=-13.77,$ *p* > 0.05). Detailed results are presented in Tables 3, 4, while the specific trend changes are illustrated in Figure 3.

### 3.3 ITS analysis of DRG reform on length of stay and hospitalization costs in tertiary TCM hospitals

We performed Cumby-Huizinga autocorrelation tests to analyze the length of stay and hospitalization costs in tertiary TCM hospitals. The results suggested that the length of stay might exhibit first-order autocorrelation, while hospitalization costs also appeared to demonstrate first-order autocorrelation, as detailed in Table 2. To ensure the validity of the ITS analysis results, we accounted for the effects of autocorrelation by employing the ‘lag (1)’ command in our analysis (see Table 5).

**Table 5:** Autocorrelation test results of length of stay and hospitalization costs in tertiary TCM hospitals.

Length of stay								Hospitalization costs							
H_0_: *q* = 0 (serially uncorrelated)				H_0_: *q* = lag-1				H_0_: *q* = 0 (serially uncorrelated)				H_0_: *q* = lag-1			
H_1_: s.c. present at range specified				H_1_: s.c. present at lag specified				H_1_: s.c. present at range specified				H_1_: s.c. present at lag specified			
lags	chi^2^	df	*p*-value	lags	chi^2^	df	*p*-value	lags	chi^2^	df	*p*-value	lags	chi^2^	df	*p*-value
1–1	13.26	1	<0.001	1	13.26	1	<0.001	1–1	17.67	1	<0.001	1	17.67	1	<0.001
1–2	13.74	2	0.001	2	0.23	1	0.634	1–2	18.24	2	<0.001	2	0.59	1	0.442
1–3	13.85	3	0.003	3	0.12	1	0.732	1–3	21.34	3	<0.001	3	1.99	1	0.158
1–4	14.00	4	0.007	4	0.33	1	0.564	1–4	22.21	4	<0.001	4	3.57	1	0.059
1–5	14.71	5	0.012	5	1.21	1	0.272	1–5	22.40	5	0.001	5	2.13	1	0.144
1–6	14.85	6	0.021	6	0.85	1	0.356	1–6	22.48	6	0.001	6	0.54	1	0.464

**Figure 4:**
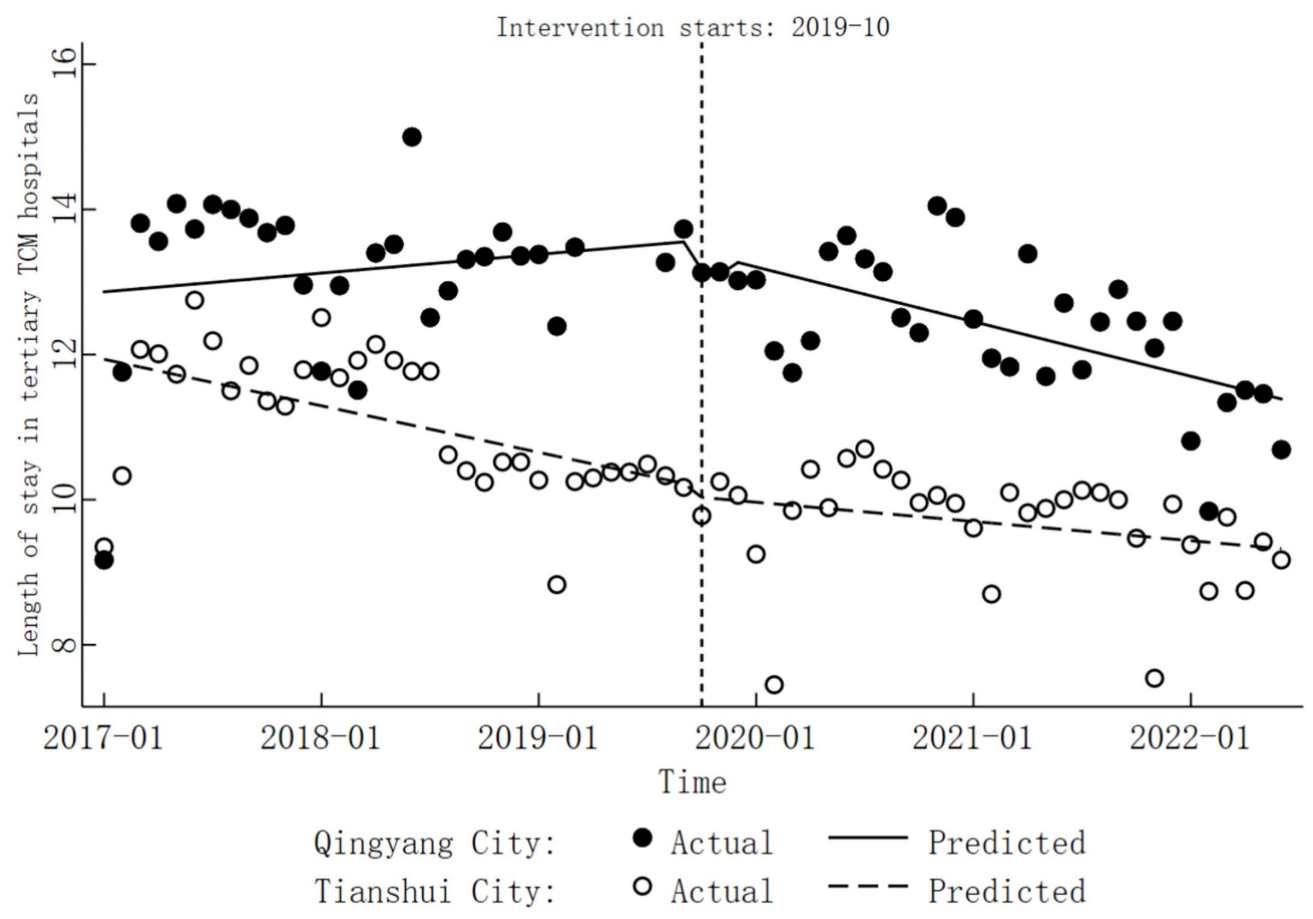
Trends in length of stay before and after the DRG reform in tertiary TCM hospitals.

In Tianshui City, the length of stay in tertiary TCM hospitals exhibited a significant decreasing trend before the implementation of DRG reform, with a monthly average reduction of 0.05 days ( $\beta_{1}=-0.05,$ *p* < 0.05). During the reform period, the length of stay did not change significantly ( $\beta_{2}=-0.14,$ *p* > 0.05), and the trend of change thereafter also remained inconclusive ( β=−0.02, *p* > 0.05). Detailed results are provided in Tables 6, 7, while specific trend changes are illustrated in Figure 4.

**Table 6:** Results of ITS analysis of length of stay and hospitalization costs in tertiary TCM hospitals.

Variables	Length of stay					Hospitalization costs				
	Coefficient	Newey-west std. err	*t*-value	*p*-value	95% conf. interval	Coefficient	Newey-west std. err	*t*-value	*p*-value	95% conf. interval
*T*	−0.05	0.02	−2.47	0.015	[−0.10, −0.01]	−19.01	7.82	−2.43	0.017	[−34.43, −3.53]
*Z*	1.16	0.88	1.32	0.189	[−0.58, 2.89]	38.64	310.06	0.12	0.901	[−575.31, 652.60]
*ZT*	0.07	0.04	1.96	0.052	[0.00, 0.15]	78.79	11.58	6.80	<0.001	[55.86, 101.73]
*X*_2019m10_	−0.14	0.45	−0.31	0.757	[−1.03, 0.75]	−81.87	188.81	−0.43	0.665	[−455.73, 291.99]
*XT*_2019m10_	0.03	0.03	1.19	0.235	[−0.02, 0.08]	−1.85	9.74	−0.19	0.850	[−21.15, 17.44]
*ZX*_2019m10_	−0.27	0.62	−0.43	0.668	[−1.51, 0.97]	−150.74	234.79	−0.64	0.522	[−615.65, 314.17]
*ZXT*_2019m10_	−0.12	0.05	−2.56	0.012	[−0.21, −0.03]	−118.40	15.74	−7.52	<0.001	[−149.56, −87.24]
COVID-19	0.23	0.37	0.62	0.539	[−0.51, 0.96]	682.80	197.14	3.46	0.001	[292.44, 1073.15]
cons	11.71	0.60	19.67	<0.001	[10.53, 12.88]	5905.90	250.58	23.57	<0.001	[5409.73, 6402.07]

**Table 7:** Trends in hospitalization costs and length of stay in tertiary TCM hospitals in Qingyang City and Tianshui City after DRG reform.

Indicators	Cities	Coefficient	Std. err.	*t*-value	*p*-value	95% conf. interval
Length of stay	Qingyang City	−0.06	0.02	−3.29	0.001	[−0.10, −0.03]
	Tianshui City	−0.02	0.01	−1.47	0.143	[−0.05, 0.01]
	Difference	−0.04	0.02	−1.68	0.096	[−0.09, 0.01]
Hospitalization costs	Qingyang City	−60.47	8.92	−6.78	<0.001	[−78.14, −42.80]
	Tianshui City	−20.86	5.84	−3.57	0.001	[−32.42, −9.30]
	Difference	−39.61	10.66	−3.71	<0.001	[−60.72, −18.49]

In the tertiary TCM hospitals of Qingyang City, the trend in length of stay before the implementation of DRG reform was not statistically significant (combined $\beta_{1}$ , $\beta_{5}$ ). During the reform period, no significant changes were observed in the levels of length of stay (combined $\beta_{2}$ , $\beta_{6}$ ). However, a significant downward trend emerged post-reform, with an average monthly reduction of 0.06 days ( β=−0.06, *p* < 0.05). Apart from this, the COVID-19 pandemic did not have a significant impact on the length of stay ( $\beta_{8}=0.23,$ *p* > 0.05). Detailed results are provided in Tables 6, 7, while specific trend changes are depicted in Figure 4.

**Figure 5:**
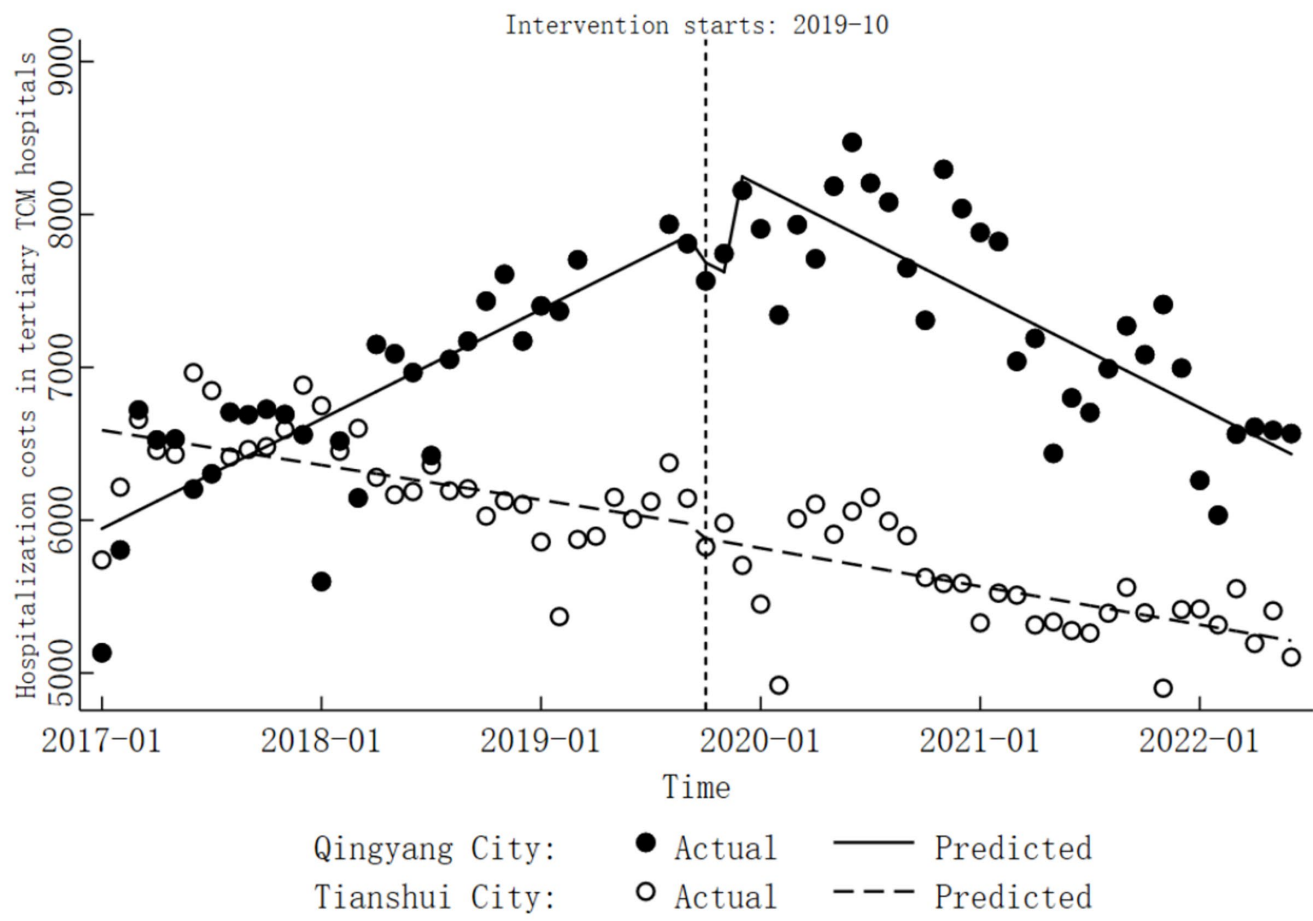
Trends in hospitalization costs before and after the DRG reform in tertiary TCM hospitals.

In Tianshui City, the hospitalization costs in tertiary TCM hospitals displayed a significant downward trend before the implementation of DRG reform ( $\beta_{1}=-19.01,$ *p* < 0.05). During the reform period, there were no significant changes in hospitalization costs ( $\beta_{2}=-81.87,$ *p* > 0.05). Post-reform, a continued significant decline in hospitalization costs was observed ( β=−20.86, *p* < 0.05). The average monthly decrease in hospitalization costs before and after the reform was relatively modest (CNY 19.01 vs. CNY 20.86). Detailed results are provided in Tables 6, 7, with specific trend changes illustrated in Figure 5.

In the tertiary TCM hospitals of Qingyang City, hospitalization costs exhibited a significant upward trend before the implementation of DRG reform (combined $\beta_{1}$ , $\beta_{5}$ ). During the reform period, hospitalization costs remained unchanged and were not statistically significant (combined $\beta_{2}$ , $\beta_{6}$ ). However, a substantial downward trend emerged post-reform, with an average monthly decrease of CNY 60.47 ( β=− 60.47, *p* < 0.05). Following the DRG reform, the monthly average hospitalization costs in these hospitals shifted from an upward trajectory to a significant decline. Apart from this, the COVID-19 pandemic contributed to the increase in hospitalization costs ( $\beta_{8}=682.80,$ *p* < 0.05). Detailed results are presented in Tables 6, 7, while specific trend changes are illustrated in Figure 5.

## 4 Discussion

This study is the first in China to empirically examine DRG-based health insurance payment reforms across different levels of TCM hospitals. Using two-group ITS models, with Tianshui TCM hospitals as the control, we compared the reform outcomes at Qingyang TCM hospitals while controlling for the confounding effects of the pandemic. Results showed that while the length of stay increased and hospitalization costs rose significantly at Qingyang’s secondary TCM hospitals post-reform, no significant changes occurred at Tianshui. Conversely, both the length of stay and hospitalization costs decreased substantially at Qingyang’s tertiary TCM hospital post-reform, with Tianshui seeing a similar hospitalization costs reduction but no change in length of stay. In summary, the DRG reform had limited effects on secondary TCM hospitals but significantly improved hospitalization costs and length of stay management in tertiary hospitals. These findings provide valuable insights for developing countries and regions reforming TCM health insurance systems to reduce the economic burden of disease.

We found that the DRG reform effectively reduces both the length of stay and hospitalization costs in tertiary TCM hospitals, aligning with existing studies (27–29). This improvement likely stems from enhanced regulation of medical practices, optimal resource utilization, and improved service quality following the implementation of the DRG payment policy. Under fixed insurance payment standards, hospital staff aims to minimize unnecessary medications, tests, and services while ensuring positive patient outcomes. Additionally, treatment protocols and services have been standardized and refined according to DRG rules, resulting in quicker patient recovery and controlled lengths of stay and costs.

Interestingly, while the DRG reform positively impacted tertiary TCM hospitals, it did not yield similar benefits for secondary TCM hospitals, where the length of stay and hospitalization costs even increased. We hypothesize that this discrepancy relates to the hospitals’ internal capabilities. Tertiary TCM hospitals generally possess advanced information technology systems that facilitate timely monitoring and feedback on non-compliant costs and practices. Moreover, the overall medical skills and adaptability of physicians in tertiary hospitals are often superior. Consequently, these hospitals can more swiftly adjust their operational mechanisms in response to the information-intensive DRG payment model, enabling physicians to rapidly modify their practices with institutional support. These findings underscore the importance of developing a hierarchical medical system and enhancing hospital information technology to optimize and promote comprehensive reforms in health insurance payments for TCM hospitals in China (30, 31). Furthermore, by consulting with experts in clinical practice, medical insurance, medical records, and hospital operations management, it has been ascertained that the differential cost-control effectiveness of DRG payment reform between tertiary and secondary traditional Chinese medicine hospitals stems from a structural contradiction between standardized payment models and differentiated service provision. This discrepancy is further amplified by disparities in implementation capacity between the two hospital tiers. Tertiary hospitals, which treat complex and severe cases, align closely with the DRG “retained savings” incentive mechanism, as their goal of cost control through enhanced efficiency fits well with the system’s design. In contrast, secondary hospitals primarily manage common and chronic diseases requiring long-term comprehensive care. The fixed DRG payment standards often fail to match their actual service costs and cycles, leading to financial strain and potentially inadequate service provision. Moreover, the pronounced advantages of tertiary hospitals in medical record front-page quality control, DRG coding expertise, and information management capabilities continue to be a critical focal point. These strengths ensure accurate case grouping and appropriate reimbursement, enabling more effective cost monitoring and clinical pathway optimization. Meanwhile, secondary hospitals’ relative weaknesses in these foundational execution areas not only compromise the accuracy of medical insurance payments but also hinder their ability to implement refined cost-control measures. Consequently, this disparity in outcomes fundamentally results from the interplay between the payment system’s design logic and the functional positioning, cost structures, and implementation foundations of hospitals at different levels.

In conclusion, the DRG-based health insurance payment reform for TCM hospitals in China has proven effective, particularly in managing the length of stay and hospitalization costs in tertiary TCM facilities, showcasing unique advantages compared to Western medicine hospital (32–34). To further enhance these reforms, a collaborative approach is essential, integrating medical services, pharmaceuticals, and health insurance. Expanding effective payment methods, such as the Diagnosis Intervention Packet (DIP) (35, 36)—which, with its relatively lower requirements for hospital informatization and operational management capabilities, remains effective in cost control and improving diagnostic-treatment efficiency, thus making it well-suited for secondary and lower-tier medical institutions—alongside strengthening the hierarchical diagnosis and treatment system will alleviate pressures and promote equitable healthcare (37, 38). Additionally, improving training quality for medical personnel and deepening the medical information management system—particularly in case documentation—will enhance the quality of real-world data within the TCM framework (39, 40). Implementing these strategies will advance health insurance payment reform in TCM hospitals, leading to better control of medical costs and improved healthcare services.

## 5 Limitations

We evaluated the effects of DRG-based health insurance payment reform for TCM hospitals in Qingyang, Gansu Province, using Tianshui City as the control group. Furthermore, as this study is based on data from two cities in Gansu Province, the sample size remains relatively limited. At the same time, due to the current unavailability of the most recent data, the long-term effects of the DRG reform in TCM hospitals have yet to be fully observed. Future research will aim to update and expand the scope to include multiple regions, while also utilizing more current data resources for validation. Moreover, the selected research indicators, particularly those related to medical costs, require further expansion. This limitation stems from the current inadequacies in TCM hospitals’ information technology infrastructure, which affects the completeness of hospital case documentation and complicates data acquisition. Nevertheless, we anticipate improvements as health insurance reform advances.

## 6 Conclusion

The DRG-based health insurance payment reform has significantly impacted TCM hospitals, particularly in controlling the length of stay and hospitalization costs in tertiary hospitals. However, the effectiveness of this reform in secondary TCM hospitals needs improvement. To advance China’s DRG reform to control the growth of health costs and ensure medical care services, it is crucial to enhance the establishment of health insurance systems, elevate the qualifications of medical personnel, and strengthen information technology infrastructure in TCM hospitals. Additionally, differentiated health insurance reform measures should be implemented for TCM hospitals at various levels to optimize medical cost control and enhance the quality of healthcare services. Specifically, the core of the differentiated DRG reform for secondary and tertiary TCM hospitals lies in “categorized payment, tiered evaluation, and targeted enablement.” Secondary hospitals can explore hybrid payment models (e.g., “DRG + hospital tier-based payment difference/primary care disease subsidy”) and more flexible performance indicators for chronic disease management, while tertiary hospitals focus on refined management based on case complexity and severity. For IT infrastructure, the key is adopting a centralized “regional cloud platform” model. This allows resource-constrained secondary hospitals to quickly access essential capabilities—such as medical record quality control and cost accounting—through shared regional data services, establishing a scalable digital foundation at a manageable cost.

## Data Availability

The original contributions presented in the study are included in the article/Supplementary material, further inquiries can be directed to the corresponding author.
